# The effect of endurance exercise on intestinal integrity in well‐trained healthy men

**DOI:** 10.14814/phy2.12994

**Published:** 2016-10-26

**Authors:** Lonneke M. JanssenDuijghuijsen, Marco Mensink, Kaatje Lenaerts, Ewa Fiedorowicz, the Protégé study group, Dorien A. M. van Dartel, Jurriaan J. Mes, Yvette C. Luiking, Jaap Keijer, Harry J. Wichers, Renger F. Witkamp, Klaske van Norren

**Affiliations:** ^1^ Wageningen Food and Biobased ResearchWageningen University and ResearchWageningenThe Netherlands; ^2^ Human and Animal PhysiologyWageningen University and ResearchWageningenThe Netherlands; ^3^ Division of Human NutritionWageningen University and ResearchWageningenThe Netherlands; ^4^ Department of SurgeryNUTRIM School of Nutrition and Translational Research in MetabolismMaastricht University Medical CentreMaastrichtThe Netherlands; ^5^ Department of BiochemistryFaculty of Biology and BiotechnologyUniversity of Warmia and MazuryOlsztynPoland; ^6^ Nutricia ResearchUtrechtThe Netherlands

**Keywords:** Amino acids, betacasomorphin‐7, citrulline, dipeptidylpeptidase‐4, exercise, intestinal permeability

## Abstract

Exercise is one of the external factors associated with impairment of intestinal integrity, possibly leading to increased permeability and altered absorption. Here, we aimed to examine to what extent endurance exercise in the glycogen‐depleted state can affect intestinal permeability toward small molecules and protein‐derived peptides in relation to markers of intestinal function. Eleven well‐trained male volunteers (27 ± 4 years) ingested 40 g of casein protein and a lactulose/rhamnose (L/R) solution after an overnight fast in resting conditions (control) and after completing a dual – glycogen depletion and endurance – exercise protocol (first protocol execution). The entire procedure was repeated 1 week later (second protocol execution). Intestinal permeability was measured as L/R ratio in 5 h urine and 1 h plasma. Five‐hour urine excretion of betacasomorphin‐7 (BCM7), postprandial plasma amino acid levels, plasma fatty acid binding protein 2 (FABP‐2), serum pre‐haptoglobin 2 (preHP2), plasma glucagon‐like peptide 2 (GLP2), serum calprotectin, and dipeptidylpeptidase‐4 (DPP4) activity were studied as markers for excretion, intestinal functioning and recovery, inflammation, and BCM7 breakdown activity, respectively. BCM7 levels in urine were increased following the dual exercise protocol, in the first as well as the second protocol execution, whereas 1 h‐plasma L/R ratio was increased only following the first exercise protocol execution. FABP2, preHP2, and GLP2 were not changed after exercise, whereas calprotectin increased. Plasma citrulline levels following casein ingestion (iAUC) did not increase after exercise, as opposed to resting conditions. Endurance exercise in the glycogen depleted state resulted in a clear increase of BCM7 accumulation in urine, independent of DPP4 activity and intestinal permeability. Therefore, strenuous exercise could have an effect on the amount of food‐derived bioactive peptides crossing the epithelial barrier. The health consequence of increased passage needs more in depth studies.

## Introduction

The intestinal epithelium is the body's largest interface between the external and internal environment, regulating fluxes of ions, water, nutrients, and other molecules while protecting the host against potentially harmful agents and invading microorganisms. The importance of a well‐functioning intestinal barrier is generally supported. At the same time, far less is understood about the nature and consequences of what is generally referred to as “increased intestinal permeability” (Bischoff et al. 2014; Quigley 2016). However, a “leaky gut” concept as persistently used in popular media as an all‐embracing cause of multiple diseases is an obvious oversimplification (Quigley 2016). In fact, “leaks” in the intestinal barrier, either discretely localized or scattered along the full length of the intestines, can relate to many distinct processes, such as an impaired mucus layer, disrupted tight junctions, an attenuated immune defense, changes in intestinal cell functionality, etc. Several studies relate impairment in absorption functions of the intestinal barrier, either intentional or unintentional, to the development of certain disorders, but knowledge on the exact mechanisms is lacking. As a consequence, a demand for a more detailed understanding of intestinal functioning remains, including insight in those processes and factors involved in impairment or restoration of its barrier function.

Particular forms of physical stress, including strenuous exercise, are known to change barrier function. Intestinal integrity can be assessed by its permeability to small compounds, such as the inert sugar lactulose and by circulating markers of intestinal damage, such as fatty acid binding protein 2 (FABP2) and prehaptoglobin 2 (preHP2) (Wang et al. 2000; Pelsers et al. 2005). Several studies, although inconclusive (Ryan et al. 1996; van Nieuwenhoven et al. 1999), have shown increased intestinal permeability to small molecules after exercise (Øktedalen et al. 1992; Pals et al. 1997; Marchbank et al. 2011; van Wijck et al. 2011a). The lack in consistent outcomes might reflect that the various exercise studies are difficult to compare, also because they differ in type and intensity of exercise as well as in the methodology to assess intestinal permeability.

The prevalence of food‐dependent exercise‐induced anaphylaxis furthermore suggests that increased absorption of intact and immunologically active proteins, or their fragments, after strenuous exercise is likely (Matsuo et al. 2005). Although it has been shown, to some extent, that specific protein and protein‐derived peptides are detectable in blood and urine after oral ingestion in humans (Castell et al. 1997; Chabance et al. 1998; Matsuo et al. 2005; Sokolov et al. 2014), limited studies have been performed on the effect of strenuous exercise on the passage of intact proteins and their fragments. This is of relevance for extrapolation to the general population, because some fragments can have specific bioactive activities or may induce adverse reactions of the immune system.

In a recent study, we used a dual exercise protocol involving both a glycogen depletion interval exercise and prolonged endurance exercise, which resulted in a clear postexercise inflammatory response in well‐trained individuals (Carol et al. 2011). In this study, this protocol was applied to study intestinal integrity and intestinal function (digestion and enterocyte metabolic mass). The casein protein bolus ingested in this study is a source of several bioactive peptides, which are known for their resistance to digestion (Segura‐Campos et al. 2011). One of the casein‐derived bioactive peptides that have been identified in vivo is betacasomorphin‐7 (BCM7). The aim of this study was to determine the effects of a controlled dual exercise protocol (Carol et al. 2011) on intestinal permeability to small molecules and excretion of protein‐derived peptides in relation to markers of intestinal function.

## Material and Methods

### Subjects

Shortly after the competition season had ended, twelve well‐trained healthy male cyclists were recruited via posters at the university, local sport centers, and via social media. Subjects were included with at least 2 years of cycling experience, a training frequency of at least two times a week during high season and no known records of milk allergy, immune diseases or intestinal diseases. Exclusion criteria were smoking, using hard drugs, using NSAIDs on a chronic basis, using any medication for gastric or intestinal complaints or donating blood during the last 6 weeks before the start and during the study. Subjects were requested not to perform intense physical activity 3 days prior to the test days. They were also instructed not to consume dairy‐containing food products 2 days prior to and during the test days, not to use alcohol 4 days prior to and during the test days, and not to use soft drugs 2 weeks prior to and during the test days.

This study was approved by the medical ethical committee of Wageningen University (METC‐WU (13/10); CCMO Number NL44947.081.13), and conducted in accordance with the Declaration of Helsinki (revised version, October 2008, Seoul). Each of the subjects gave their written informed consent.

### Baseline testing

Baseline testing was performed in the week prior to the start of the experimental protocol and consisted of anthropometric measurements including DEXA scan and a maximal aerobic capacity test. The maximal aerobic capacity test was adapted from (Storer et al. 1990) and performed using an electronically braked cycle ergometer (Lode Excalibur, Groningen, The Netherlands). After a short warming‐up, the subjects started cycling at 100 W. Every minute the power increased with 20 W. The subjects had to cycle until they were no longer able to maintain the workload (W_max_; pedal frequency falling from 90 to 100 rpm to <70 rpm). O_2_‐consumption and CO_2_‐production were measured with an Oxycon Pro (Jaeger, Hoechberg, Germany) to define the maximal aerobic capacity, VO_2max_.

### Study design

**Figure 1:**
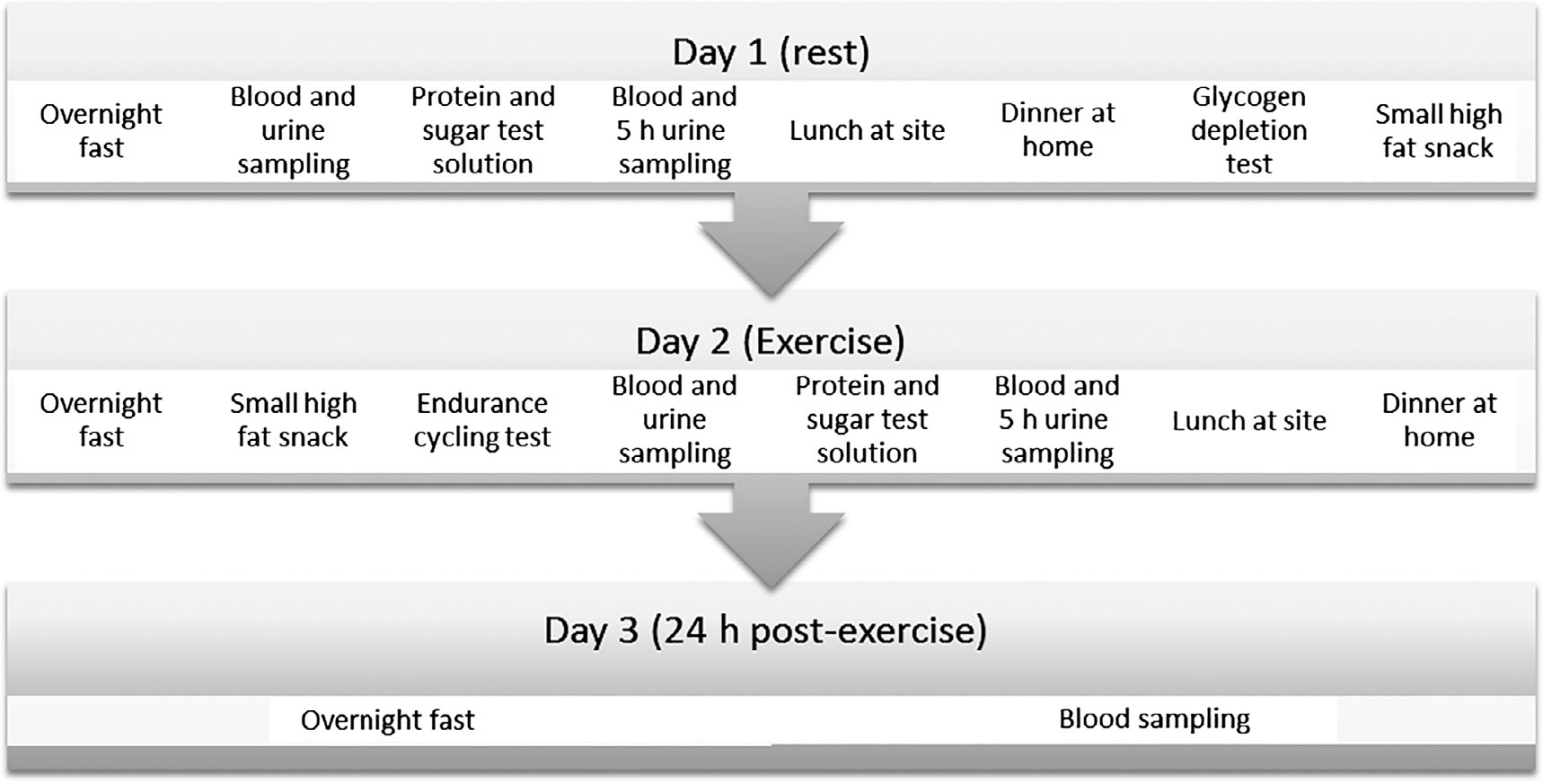
Schematic overview of experimental design. This scheme shows the different stages of the 3‐day experimental protocol (first protocol execution). This complete experimental protocol was repeated after 1 week in exactly the same way (second protocol execution).

**Figure 2:**
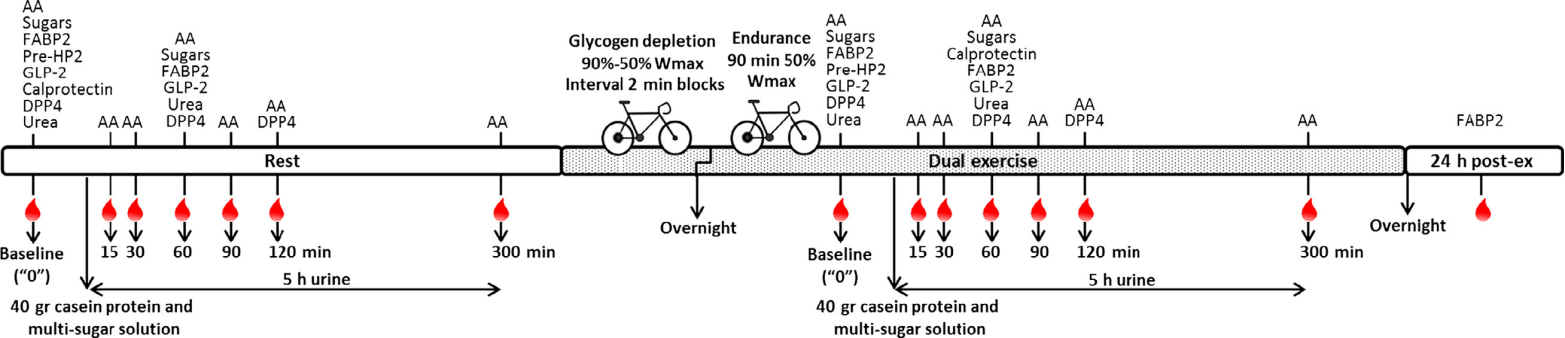
Schematic overview of blood sampling and analyses during one repetition of the experimental protocol. This scheme shows the blood sampling during rest, exercise, and 24 h post exercise. The resting condition started at 0800 h and finished around 1400 h with lunch. The exercise condition started around 2000 h in the evening of the same day (2 h after dinner), and continued at 0730 h the next morning, and sampling lasted till around 1400 h on that day. The 24 h post exercise blood sampling took place around 0900 h the next day. This complete experimental protocol with a dual exercise protocol was repeated in exactly the same way 1 week later. Samples taken at baseline were used as *t* = 0 for calculations. The different blood analyses are displayed at the different sampling time points. AA: amino acids; sugars: lactulose, l‐rhamnose, and d‐xylose.

This study had a single arm intervention design, with measurements performed during three conditions: rest, exercise, and 24 h post exercise (see Figs 1 and 2). Each subject acted as their own control (resting condition).

The experimental protocol started with assessing intestinal permeability during the resting condition. The subjects arrived at the university after being fasted overnight (since 2300 h). A venflon cannula was inserted in the antecubital vein and blood was sampled (baseline‐rest, taken as *t* = 0 for calculations). A baseline urine sample was also collected. This was followed by the ingestion of a multi‐inert‐sugar test solution (see intestinal permeability testing) and a protein bolus containing 40 g of casein protein (Kind gift of Dutch Protein & Services BV, Tiel, The Netherlands) dissolved in 400 mL of tap water. Blood and urine was sampled (with *t* = 15 being 15 min after ingestion of casein) during the next 5 h at regular intervals, after which lunch was offered. The 5 h duration of sampling was chosen to accommodate possible slow digestion kinetics of casein (Boirie et al. 1997; Tang et al. 2009).

The exercise condition consisted of a dual exercise protocol and started in the evening following the assessment of the resting control condition. The subjects returned to the university 2 h after their regular dinner at home and completed the first part of the dual exercise intervention. This first part aimed at achieving glycogen depletion and consisted of 2‐min blocks of cycling alternating between very high intensity (90% W_max_ ≈ 90% VO_2max_), and moderate intensity (50% W_max_ ≈ 55% VO_2max_) at a pedal frequency of 90–100 rpm (Arts and Kuipers 1994; Pollock et al. 1998). When they were no longer able to cycle at 90% W_max_, the intensity was reduced to 80% W_max_ (still alternating with 50% W_max_), and subsequently to 70% W_max_. When alternating 70% and 50% W_max_ could not be maintained, this exercise protocol was stopped. The subjects then received a small high‐fat snack (Two crackers with peanut butter: 164 kCal of which 53 energy percent (En%) fat and 26 En% carbohydrates; or two crackers with pate: 260 kCal of which 61 En% fat and 32 En% carbohydrates) to reduce hunger feelings at night. They were not allowed to eat anything else until returning to the university the next morning, where they received the same high‐fat snack before continuing with the second part of the dual exercise intervention. This second part consisted of 90 min of cycling at a moderate intensity of 50% W_max_, which can be considered strenuous in combination with the prior glycogen depletion part. Directly after completing the second part of the dual exercise protocol, a venflon cannula was inserted in the antecubital vein and blood was sampled (baseline‐ exercise, taken as *t* = 0 for calculations). A baseline urine sample was also collected. This was again followed by the ingestion of the multi‐inert‐sugar test solution and the casein protein solution. Blood and urine was sampled (with *t* = 15 being 15 min after ingestion of the casein protein solution) for 5 h at regular intervals, after which lunch was offered.

The next morning, the subjects came back to the university once more (24 h post exercise condition) to donate a single fasted blood sample.

This complete single arm experimental protocol was repeated after 1 week to test for reproducibility in outcomes. In the following sections, these two executions of the experimental protocol are referred to as first and second protocol execution.

### Blood and urine sampling

Venous blood was sampled during rest and exercise at baseline and at 15, 30, 60, 90, 120, and 300 min after casein protein intake. In the two cases that the venflon catheter could not be successfully placed, blood was sampled by venapunction at baseline and at 60, 120, and 300 min after casein protein intake. One fasted blood sample was drawn by venapunction 24 h post exercise. Blood for the analyses was sampled in tubes (Vacutainer; Becton Dickinson, Breda, The Netherlands) containing either EDTA or EDTA supplemented with protease inhibitor cocktail (Sigma Aldrich, Zwijndrecht, The Netherlands), which were directly centrifuged at 2000 *g* for 10 min at 4°C, aliquoted and stored at −80°C within 20 min after sampling until further analysis. Blood was also sampled in serum separator tubes; it was left to clot in the dark for at least 30 min at room temperature, after which the tubes were centrifuged at 2000 g for 10 min at room temperature to obtain serum, which was directly aliquoted and stored at −80°C until further analysis.

Collected urine was supplemented with chlorhexidine to prevent bacterial growth. During both the resting and the exercise condition, one urine sample was collected at baseline and urine was collected and pooled over a 5‐h period following intake of the multi‐inert‐sugar and casein bolus. The total sample volume was recorded and aliquots were stored at −20°C until further analysis.

### Parameters of intestinal permeability

To study intestinal permeability to inert, nondigestible sugar molecules, a multi‐sugar test solution was used containing three sugar probes: 5 g of lactulose, 1 g of l‐rhamnose, and 0.5 g of d‐xylose (BCM Specials, Nottingham, UK). These sugars were dissolved in 100 mL tap water and ingested at the start of the resting condition as well as the exercise condition. To analyze urinary levels of lactulose, l‐rhamnose, and d‐xylose, 250 *μ*L urine was diluted 20 times with 0.08 mmol/L AgNO_3_ to precipitate chloride, after which the supernatant was desalted by eluting it over a Dionex Onguard Ba and H column (Thermo Fisher Scientific BV, Breda, The Netherlands). This eluate was analyzed by HPAEC (Dionex‐HPLC ICS5000; Thermo Fisher Scientific) using pulsed electrochemical detection. Urinary sugar probe excretion was calculated by multiplying the concentration of each of the sugars probes with urine volume, after which the baseline excretion was subtracted. Recovery of the sugar probes was expressed as percentage of the orally ingested dose. Lactulose‐to‐l‐rhamnose (L/R) ratios were calculated from the urinary excretion levels. An increased L/R ratio implies increased intestinal permeability. Levels of lactulose, l‐rhamnose, and d‐xylose were also measured in EDTA plasma. 125 *μ*L EDTA plasma was transferred to Eppendorf tubes containing a 3000 Da cut‐off filter (Amicon Ultra 0.5 mL 3 K; Millipore) to remove the plasma proteins. The filter tubes were centrifuged for 30 min at 11,000 *g* at 4°C, and clear plasma filtrate was inserted in the cooled sample processor (233 XL; Gilson, Middleton, WI). Sugars were subsequently analyzed by LC‐MS as described elsewhere (van Wijck et al. 2011b). Plasma L/R ratios were calculated from the 1 h plasma concentrations corrected for the concentrations found at the respective baseline.

### Betacasomorphin‐7 and dipeptidylpeptidase‐4

To measure intestinal passage of protein‐derived peptides, a specific fragment of casein, betacasomorphin‐7 (BCM7), was measured in baseline and 5 h urine samples, cleaned‐up by centrifugation through a 30 kD cut‐off filter (Amicon Ultra; Merck Millipore, Co. Cork, Ireland). Flat‐bottom ELISA plates (MaxiSorp, Nunc, Roskilde, Denmark) were coated with 100 *μ*L 1.5 *μ*g/mL BCM7‐polylysine conjugate solution. The analysis was performed in duplicate with the competitive ELISA described elsewhere (Sienkiewicz‐Szłapka et al. 2009), in which the primary anti‐BCM7 polyclonal antibodies (Abnova, Taipei, Taiwan) were used in concentration of 3.4 *μ*g/mL.

Dipeptidylpeptidase‐4 (DPP4; EC 3.4.14.5) is the only enzyme able to breakdown BCM7 (Kreil et al. 1983). Therefore, serum activity of DPP4 in sample taken at baseline, as well as 60 and 120 min after casein intake, were determined for its potential effect on urine BCM7 levels. The photometric method was used as described by Wasilewska et al. (2011). The calculations were made after adjusting the measurements with the blank; the enzyme activity was calculated as: 100 × (*E* − *C*)/*S* (where *E*,* C,* and *S* stand for the absorbance of the test, control, and standard samples, respectively). One unit of the enzyme activity was defined as the amount of the enzyme liberating 1 *μ*mol *p*‐nitroanilide/min/L test serum at 37°C.

### Intestinal damage and recovery markers

FABP2 was measured as an acute marker of small intestinal injury in EDTA plasma (1:1) with an in‐house developed ELISA, as described elsewhere (Van Wijck et al. 2012).

Serum prehaptoglobin‐2 (preHP2), also known as zonulin, was measured as a marker of intestinal integrity in serum (1:20) with a commercial ELISA kit (Immundiagnostik AG, Bensheim, Germany), following the instructions of the manufacturer. This ELISA kit only detects the active, uncleaved, form of preHP2.

Plasma glucagon‐like peptide 2 (GLP2) [1–34] levels were measured because this compound is positively related to improvement of intestinal permeability (Benjamin et al. 2000). GPL2 was measured in duplicate in EDTA plasma treated with protease inhibitor using a commercial ELISA kit (Phoenix pharmaceuticals, Karlsruhe, Germany) according to instructions of the manufacturer. Prior to testing, 200 *μ*L of plasma was loaded onto a pretreated C18 SEP‐column (Phoenix Pharmaceuticals) to remove proteins, after which the eluent was freeze‐dried. The lyophilized samples were reconstituted in 125 *μ*L assay buffer.

### Inflammatory marker

Serum calprotectin is an antimicrobial protein considered to be an early parameter of systemic inflammation (Mortensen et al. 2008). Calprotectin analyses were performed with the EliATM Calprotectin assay following manufacturer's instructions (14‐5610‐01; Thermo Fisher Scientific, Etten‐Leur, The Netherlands) by a specialized medical laboratory (SHL, Etten‐Leur, The Netherlands).

### Amino acid and urea levels

Plasma amino acids (AAs) were analyzed with HPLC after precipitating plasma proteins in 50 *μ*L plasma with 200 *μ*L 3% perchloric acid. Individual AAs were determined by UFLC (Shimadzu, 's‐Hertogenbosch, The Netherlands) using a precolumn derivatization with o‐phtaldialdehyde and fluorimetric detection (Terrlink et al. 1994). Urea is a primary metabolite derived from dietary protein and was measured in serum (1:100) with a commercial assay (Abcam, Cambridge, UK).

### Statistics

Repeated measures ANOVA with Tukey's multiple comparison tests were used to assess changes in plasma GLP2, serum and plasma FABP2, serum preHP2 in response to exercise (exercise condition) and casein intake only (resting condition) (GraphPad Prism 6, GraphPad Software Inc., San Diego, CA).

Ratio paired *T*‐tests were used to assess changes in urinary and plasma sugars probes, L/R ratios, urinary BCM7 and serum calprotectin between resting and exercise condition (GraphPad Prism 6). When urinary BCM7 levels were too low to detect in the urine, a lower level of detection (1.0 ng/mL) was imputed to enable paired statistics.

Exercise, protocol execution, and exercise × protocol execution were the fixed parameters of the mixed model with LSD estimated marginal means which was used to assess changes in AA levels and AA‐level‐derived incremental area under the curve (iAUC) in response to exercise and casein intake (IBM SPSS Statistics 22, IBM Corporation, Armonk, NY).

As per protocol analysis was performed. One subject was excluded because this subject had not been following the dietary guidelines with regard to fasting. This subject was taken out of all calculations. Some individuals were excluded from analysis when there were missing blood samples. The actual number of subjects included in each of the analysis was 11, unless stated otherwise in the figure caption. Statistical significance was defined as a two‐tailed *P* < 0.05.

## Results

### Subject characteristics

Physical parameters of the 11 male volunteers are shown in Table 1.

**Table 1:** Subject characteristics

Age (years)	26.7 ± 3.5
Height (m)	1.87 ± 6.5
Weight (kg)	77.8 ± 9.4
BMI (kg/m^2^)	22.2 ± 1.5
Fat mass (% of total mass)	13.6 ± 6.1
W_max_ (W)	406.4 ± 25.4
VO_2max_ (mL/kg/min)	62.5 ± 6.0

Data are shown as mean ± SD, *n* = 11.

### Exercise‐induced intestinal damage

**Figure 3:**
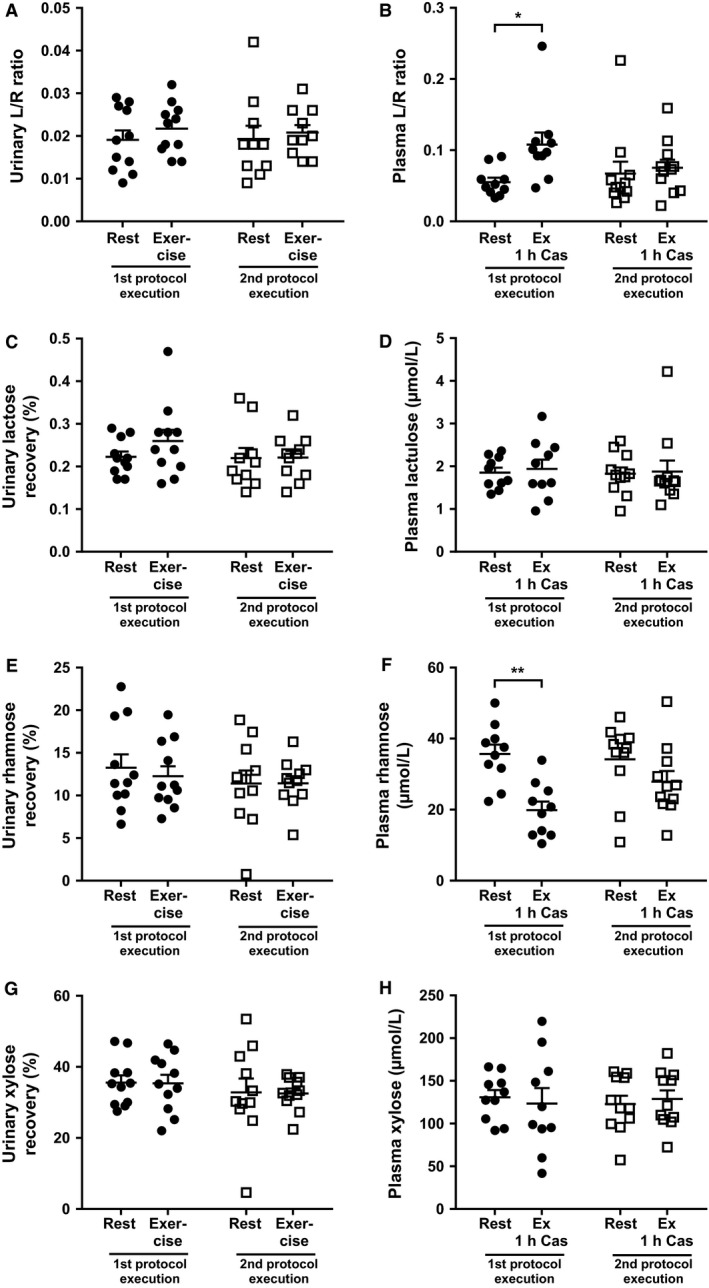
Effect of exercise on markers of intestinal permeability in urine and plasma. L/R ratio (A) and recoveries of lactulose (C), l‐rhamnose (E), and d‐xylose (G) in 5 h pooled urine are shown as mean ± SEM. L/R ratio (B) and levels of lactulose (D), l‐rhamnose (F), and d‐xylose (H) in 1 h plasma (*n* = 10 for first protocol execution) are shown as  ± SEM. Statistical comparisons are made between values during the resting condition (rest) and the exercise condition (Exercise and Exercise+ 1 h casein protein intake) within the first protocol execution (circles) and the second protocol execution (squares). The * symbol depicts *P* < 0.05 and the ** symbol depicts *P* < 0.01.

To assess exercise‐induced effects on intestinal permeability, urinary as well as plasma recovery levels of lactulose and l‐rhamnose were determined and calculated as L/R ratios. Five hour urinary lactulose, l‐rhamnose, and L/R ratios were not different after exercise compared with resting conditions (Fig. 3). In contrast, plasma L/R ratio was increased 1 h after exercise (*P* = 0.01), suggesting an increase in intestinal permeability, mainly explained by a decrease in plasma l‐rhamnose (*P* = 0.002). However, this was only the case during the first session. During the second protocol execution, there was no change in plasma l‐rhamnose and L/R ratio, suggesting no change in intestinal permeability. Urinary and plasma levels of xylose were similar for all conditions.

### Urinary BCM7 levels

**Figure 4:**
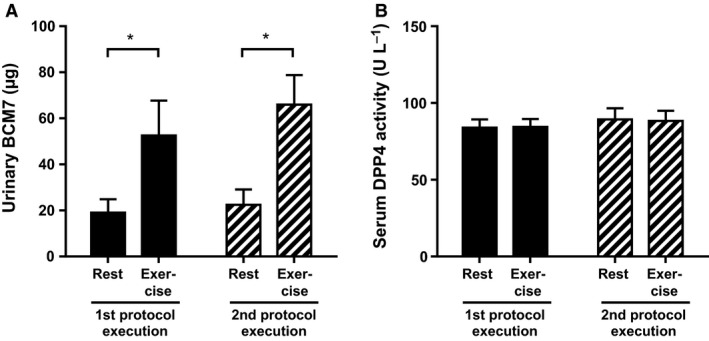
Effect of exercise and serum DPP4 activity on urine betacasomorphin‐7 (BCM7) levels. Urinary BCM7 levels (A) and serum DPP4 activity (B) (*n* = 10) are shown as mean ± SEM. Statistical comparisons are made for the first protocol execution (black) and the second protocol execution (striped) between the resting condition (rest) and the exercise condition. The * symbol depicts *P* < 0.05.

Urinary levels of BCM7 were increased after ingestion a single bolus of casein protein during the exercise condition compared to the resting condition (*P* = 0.03 for both protocol executions) (Fig. 4A), suggesting increased intestinal absorption. These effects were similar for the first and second protocol execution. Serum DPP4 activity was measured, as that can have an effect on circulating BCM7 levels. There was no effect of casein protein intake on DPP4 activity and therefore average activity (baseline, *T* = 60 and *T* = 120) for the resting condition compared to the exercise condition are shown. The levels of this peptidase were similar for each of the conditions (Fig. 4B) and individual BCM7 and DPP4 activity levels were not correlated (*R* ^2^ = 0.024, *P* = 0.38).

**Figure 5:**
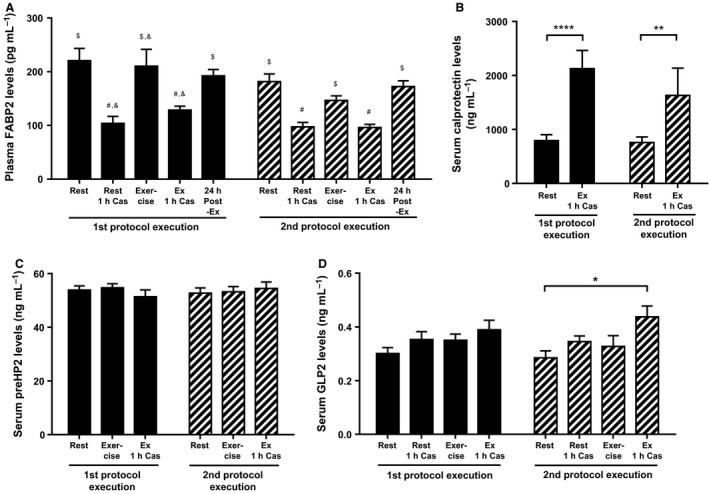
Effect of exercise on markers of intestinal integrity, recovery and systemic inflammation. Levels of plasma FABP2 (A), serum calprotectin (B), serum prehaptoglobin 2 (preHP2) (C), and serum glucagon‐like peptide 2 (GLP2) (D) are shown as mean ± SEM. Statistical comparisons are made for the first protocol execution (black) and the second protocol execution (striped) between the resting condition (rest) and the exercise condition with or without 1 h casein intake (Ex 1 h Cas), and for plasma FABP2 also 24 h post exercise. For serum preHP2, 1 h casein sample in the resting condition was not analyzed. The * symbol depicts *P* < 0.05, **P < 0.01, and ****P < 0.0001. For plasma FABP2, bars with different symbols (#, & and $) are statistically different.

The effect of exercise on small intestinal epithelial damage was determined by assessing levels of circulating FABP2. Figure 5A shows that plasma FABP2 levels were not increased directly after exercise compared to the resting condition, suggesting no intestinal epithelial damage had taken place. During both protocol executions, however, FABP2 levels decreased significantly 1 h after casein intake, independent of exercise, and in both protocol executions those levels increased again to resting levels during the exercise condition and 24 h post exercise. In line with this finding, there was no change in serum preHP2 levels when comparing the resting condition with the exercise condition (Fig. 5C). There was, however, a significant increase (First protocol execution *P* < 0.0001 and second protocol execution *P* = 0.008) in serum calprotectin levels 1 h after completing the dual exercise protocol (Fig. 5B) compared to resting condition baseline levels, indicating the presence of inflammation. During the second protocol execution, serum GLP2, a hormone that is involved in intestinal repair, was increased after the combination of exercise and casein compared to resting condition baseline values (Fig. 5D).

### Plasma AA profiles

**Figure 6:**
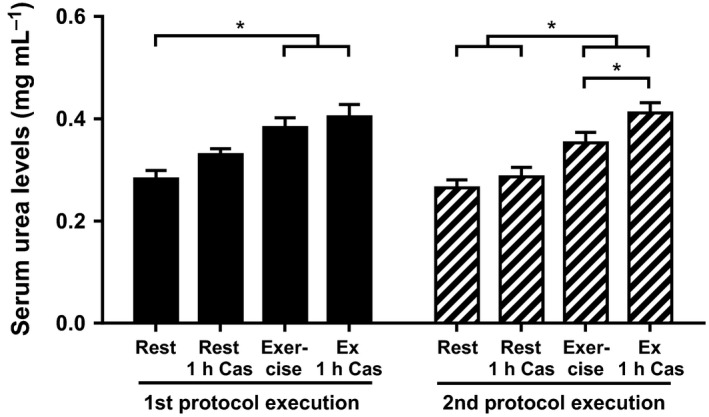
Effect of exercise and casein intake on serum urea levels. Data are shown as mean ± SEM. Serum levels during the first (black) and second (striped) protocol execution are compared between the resting condition (rest) and exercise condition in combination with 1 h casein intake (rest and Ex 1 h Cas). The * symbol depicts *P* < 0.05.

**Figure 7:**
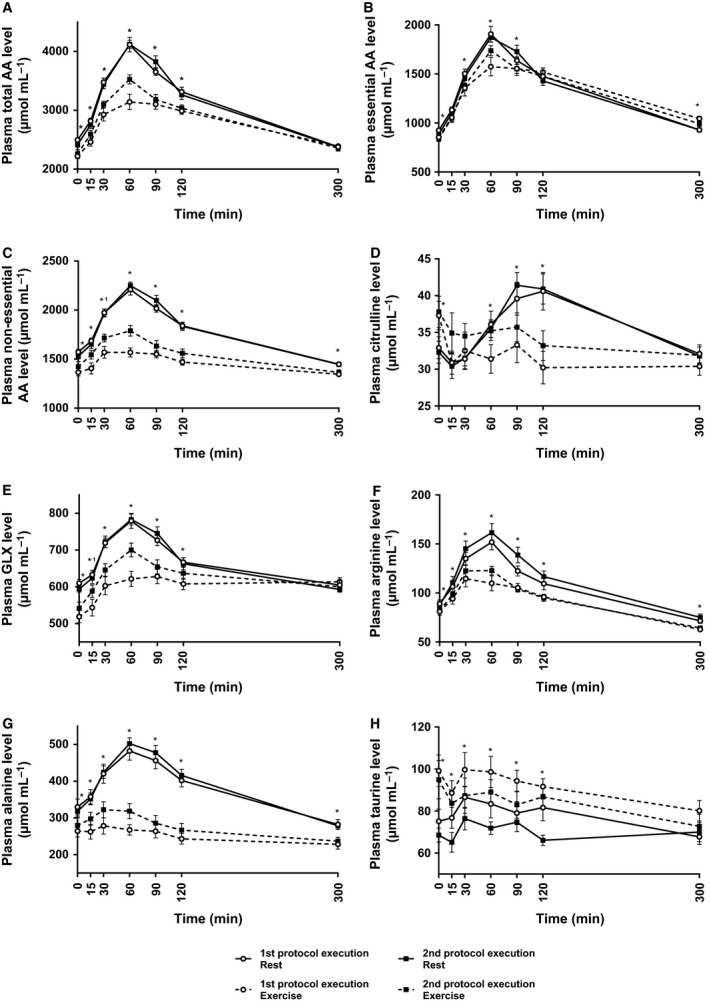
Effect of exercise on postprandial plasma amino acid levels. Plasma levels of total AAs (A), EAAs (B), nEAAs (C), citrulline (D), glutamine+ glutamate (GLX; E), arginine (F), alanine (G), and taurine (H) following casein bolus intake are shown as mean ± SEM. Comparisons are made for the first (open circles) and second (closed squares) protocol execution between the resting condition (line) versus exercise condition (dashed line). N = 10 for first protocol execution. Significant differences are depicted with * when different for both protocol executions and *^1^ when different for the first protocol execution only.

A single bolus of 40 g casein protein was ingested during the resting condition as well as during the exercise condition. Data on all AAs are in Table 2. As an effect of exercise, serum urea levels increased compared to the levels in the resting condition (Fig. 6), suggesting increased AA oxidation or breakdown under these circumstances. Absolute plasma taurine levels were constantly higher during the exercise condition compared to resting conditions (Fig. 7H).

**Table 2:** Effect of exercise on postprandial amino acid levels (as 5 h iAUC, peak height and time‐to‐peak)

		iAUC^a^ (*μ*mol min/mL; in thousands)						Maximum peak height (*μ*mol/mL)						Time‐to‐peak (min)					
		1st protocol execution			2nd protocol execution			1st protocol execution			2nd protocol execution			1st protocol execution			2nd protocol execution		
		Rest	Exercise	*P*‐value	Rest	Exercise	*P*‐value	Rest	Exercise	*P*‐value	Rest	Exercise	*P*‐value	Rest	Exercise	*P*‐value	Rest	Exercise	*P*‐value
TotAA	Mean	192.6	168.9	0.248	211.5	179.2	0.005	1668.3	1047.6	0.001	1708.6	1317.2	0.002	63.3	76.7	0.169	66.0	63.0	0.591
	SEM	20.8	14.9		11.6	15.5		135.0	104.4		87.6	116.2		3.3	8.8		4.0	5.4	
EAAs	Mean	127.9	144.2	0.206	135.7	146.1	0.009	1001.1	817.5	0.036	1009.3	933.4	0.136	63.3	76.7	0.225	72.0	66.0	0.555
	SEM	14.0	11.0		8.8	7.4		87.4	72.6		60.7	70.6		3.3	8.8		4.9	7.5	
nEAAs	Mean	66.7	27.7	0.001	77.8	39.1	<0.001	667.1	258.4	<0.001	712.1	393.1	<0.001	63.3	60.0	0.760	63.0	49.5	0.041
	SEM	7.6	5.5		4.6	6.1		54.2	39.2		36.7	53.7		3.3	11.2		3.0	7.1	
CITR	Mean	1.2	0.1	0.004	1.3	0.5	0.066	9.7	2.6	0.002	10.0	5.3	0.170	98.3	60.0	0.397	105.0	37.5	0.003
	SEM	0.3	0.1		0.3	0.2		1.8	1.3		2.1	2.5		11.5	33.5		5.0	15.0	
GLX^b^	Mean	18.0	25.8	0.090	20.8	24.7	0.177	179.8	138.5	0.113	190.2	164.3	0.057	63.3	116.7	0.173	66.0	60.0	0.343
	SEM	3.2	2.0		2.3	3.1		20.5	7.0		14.5	19.5		3.3	35.2		4.0	4.5	
ALA	Mean	17.4	2.0	<0.001	21.6	4.5	<0.001	158.7	28.6	<0.001	185.1	61.3	<0.001	63.3	70.0	0.834	69.0	42.0	0.010
	SEM	2.2	0.6		2.3	1.2		12.9	5.4		10.7	10.9		3.3	30.0		4.6	6.6	
ARG	Mean	5.9	3.7	0.053	8.0	3.8	<0.001	64.8	36.9	0.001	75.1	47.5	0.005	60.0	50.0	0.347	57.0	46.5	0.173
	SEM	0.9	0.5		0.7	0.2		6.9	4.1		7.3	2.7		0.0	10.0		3.0	5.7	
GLY	Mean	2.7	0.6	0.005	4.0	1.3	<0.001	42.6	14.8	0.005	62.5	29.9	0.008	56.7	25.0	0.007	60.0	36.0	0.002
	SEM	0.5	0.2		0.4	0.3		6.0	5.3		8.3	6.6		6.0	9.4		0.0	5.6	
ASX	Mean	4.4	2.3	0.011	4.5	3.0	<0.001	46.0	18.7	<0.001	44.9	28.7	0.001	63.3	73.3	0.397	66.0	55.5	0.173
	SEM	0.7	0.2		0.3	0.4		4.8	2.7		3.0	3.2		3.3	11.3		4.0	7.8	
HIS	Mean	2.4	3.1	0.242	2.8	3.2	0.181	29.5	21.2	0.040	30.9	28.2	0.101	63.3	70.0	0.559	60.0	57.0	0.591
	SEM	0.5	0.3		0.3	0.5		3.9	2.1		2.3	3.0		3.3	11.2		0.0	5.4	
ILE	Mean	15.8	24.9	<0.001	15.6	22.8	<0.001	128.5	137.8	0.365	128.9	140.5	0.053	63.3	83.3	0.050	75.0	69.0	0.343
	SEM	1.8	1.7		1.2	0.9		11.4	10.0		9.7	10.3		3.3	9.7		5.0	4.6	
LEU	Mean	28.5	41.6	0.002	28.8	38.2	<0.001	223.7	225.6	0.921	222.4	237.0	0.169	63.3	80.0	0.139	72.0	72.0	1.000
	SEM	2.9	2.7		1.8	1.7		20.5	17.3		13.5	16.7		3.3	11.2		4.9	6.6	
METH	Mean	5.5	1.0	<0.001	5.9	2.6	<0.001	44.4	13.4	<0.001	45.3	27.7	<0.001	63.3	43.3	0.022	69.0	54.0	0.052
	SEM	0.6	0.3		0.4	0.4		3.9	3.1		2.6	3.3		3.3	7.3		4.6	4.0	
LYS	Mean	20.5	15.2	0.028	23.5	16.3	<0.001	186.2	113.9	0.003	190.4	138.0	0.002	56.7	66.7	0.471	60.0	54.0	0.168
	SEM	2.9	1.9		1.8	1.3		19.7	14.5		12.9	10.2		3.3	10.9		0.0	4.0	
PHEALA	Mean	4.6	2.9	0.023	5.1	4.0	0.005	41.6	25.4	0.001	42.9	35.9	0.050	60.0	70.0	0.397	66.0	57.0	0.279
	SEM	0.5	0.4		0.3	0.4		3.4	3.1		2.0	3.4		0.0	11.2		4.0	5.4	
SER	Mean	8.0	3.4	0.012	6.9	4.3	0.043	71.6	29.9	0.003	69.0	39.9	0.011	66.7	70.0	0.782	69.0	54.0	0.096
	SEM	1.3	0.6		0.6	0.7		9.9	5.8		4.8	6.3		6.7	8.7		4.6	7.5	
TAU	Mean	2.0	0.8	0.024	1.2	0.4	0.047	20.4	11.9	0.092	16.5	7.3	0.062	53.3	93.3	0.311	75.0	43.5	0.299
	SEM	0.7	0.3		0.5	0.3		4.7	3.1		4.9	2.5		12.8	28.9		26.8	12.3	
THRE	Mean	10.5	6.0	0.008	10.9	7.6	0.001	85.3	42.0	<0.001	86.6	57.0	0.001	63.3	76.7	0.225	69.0	63.0	0.343
	SEM	1.5	0.6		0.8	0.7		8.0	5.1		5.8	5.3		3.3	10.1		4.6	5.4	
TRP	Mean	3.1	3.8	0.423	3.2	5.1	0.017	29.8	28.6	0.820	30.1	36.1	0.060	63.3	66.7	0.729	60.0	54.0	0.343
	SEM	0.4	0.7		0.3	0.5		2.0	4.6		2.3	1.9		3.3	8.3		0.0	6.0	
TYR	Mean	13.0	5.1	<0.001	14.0	7.9	<0.001	90.2	43.2	<0.001	92.6	60.9	<0.001	63.3	73.3	0.397	72.0	57.0	0.052
	SEM	1.2	0.7		1.1	0.6		7.7	7.0		6.6	4.7		3.3	10.1		4.9	5.4	
VAL	Mean	38.8	47.4	0.020	40.7	47.5	<0.001	238.5	234.5	0.818	252.8	254.1	0.845	70.0	90.0	0.022	75.0	87.0	0.223
	SEM	3.4	3.4		2.7	2.3		18.3	18.4		18.8	18.7		5.0	8.7		5.0	8.3	

*N* = 10 for first protocol execution; *N* = 11 for second protocol execution.

a iAUC in (*μ*mol min/mL) reflects the incremental area under the curve (in thousands) over a period of 5 h following casein protein bolus intake.

b GLX is the cumulative of glutamine and glutamic acid levels.

The postprandial increase in total AA due to casein protein intake was diminished after exercise (iAUC; *P* = 0.014) (Fig. 7A). This effect is mainly explained by a less pronounced increase in nonessential amino acids (nEAA) (iAUC; *P* < 0.001) and partly by a smaller increase in plasma essential amino acids (EAAs) (iAUC; *P* = 0.044) (Fig. 7B and C). Within the group of nEAA, alanine had the largest contribution to the diminished increase in plasma levels (Fig. 7G). A striking effect of exercise was found on the citrulline levels after casein intake (Fig. 7D). During the exercise condition, the increase in citrulline was virtually absent (iAUC, *P* < 0.001), with a 10‐fold smaller iAUC compared to resting during the first protocol execution (1216* μ*mol min/mL vs. 120 *μ*mol min/mL) and an almost threefold lower iAUC compared to resting during the second protocol execution (1341 *μ*mol min/mL vs. 488 *μ*mol min/mL) (Table 2). Citrulline can be produced by the intestine from glutamine. The cumulative plasma levels of glutamine and glutamate (GLX), however, did not show a significantly lower postprandial iAUC after exercise (Fig. 7E). Citrulline can undergo renal conversion into arginine (Fig. 7F). As for citrulline, total plasma levels of arginine were lower during the exercise condition compared to the resting condition (Table 2).

## Discussion

### Passage of betacasomorphin‐7 (BCM7)

Despite an apparently small increase in sugar permeability after exercise as seen from the classical L/R sugar test, exercise clearly increased the excretion of the casein‐derived peptide, BCM7, in 5 h urine. The urinary concentrations of this fragment were elevated after exercise during both protocol executions. These findings indicate that the intestinal passage of proteins and (or) their fragments‐ peptides is more prone to effects of exercise compared to the absorption of sugars, which may be due to different mechanisms being involved. The appearance of these peptides is probably not facilitated via the paracellular pathway, as the absence of changes in lactulose indicates unchanged paracellular transport. Decreased l‐rhamnose and unchanged d‐xylose, respectively, indicate a decreased or unaltered, but not increased, noncarrier mediated transcellular transport. Finally, the increase in BCM7 cannot be directly explained by a difference in breakdown by dipeptidylpeptidase‐4 (DPP4), because there was a similar systemic DPP4 activity in both conditions. BCM7 is a bioactive peptide which can act as an opioid receptor agonist. BCM7 has also been implicated in several beneficial and adverse health effects, reviewed in detail by the European Food Safety Authority (EFSA 2009) and Haq et al. (2014). Almost all studies on the effects of BCM7, however, have been performed in vitro or with animal models. Evidence of health effects of BCM7 in humans is limited to epidemiological data. The EFSA therefore concluded that a causal relationship between BCM7 exposure and chronic disease risk could not be supported by the existing data. Our data provide an answer to one of the knowledge gaps presented by the EFSA (2009) by showing that casein‐derived peptides can indeed be transferred intact across the intestinal barrier. We have also attempted to measure levels of casein and casein‐derived peptides in the plasma with a casein and beta‐casein sandwich ELISA as well as HPLC MS/MS techniques, but we were unable to obtain the required detection sensitivity and specificity. Endurance exercise in the glycogen depleted state resulted in increased levels of BCM7 in urine after casein consumption, but whether and how these levels could subsequently amplify either beneficial or adverse health effects, requires further investigation.

### Effects on intestinal integrity

Our dual exercise protocol did not induce similar effects on aspects of intestinal integrity as was found in previous studies. For example, FABP2, an acute enterocyte damage marker, was not affected in this study, while van Wijck et al. (2011a) found a clear increase in plasma FABP2 levels during and directly after exercise. The cause for the initial decrease in FABP2 levels following 1 h of casein intake in this study has recently been investigated. We figured out that cycling, the usual way of transportation, in a fasted condition to the test location already results in increased levels of FABP2 (L. M. JanssenDuijghuijsen, J. Keijer, M. Mensink, K. Lenaerts, L. Ridder, S. Nierkens, S. W. Kartaram, M. C. M. Verschuren, R. H. H. Pieters, R. Bas, R. F. Witkamp, H. J. Wichers, and K. van Norren, unpubl. data). This may explain our results, as a baseline blood sample was drawn directly after arrival at the test location. The FABP2 level determined after 1 h rest likely reflects the “actual” individuals' baseline level. No additional effect of the endurance exercise was found. This may be due to the lower cycling intensity of 50% W_max_ used in our protocol compared to 70% W_max_ in van Wijck et al. (2011a), as data were obtained with the same assay. It should be noted, however, that the exercise protocol used in this study can still be regarded as intensive, which is for example reflected by the release of calprotectin from skeletal muscle (Mortensen et al. 2008). Another explanation could be that the intensive glycogen depletion exercise phase the evening before the endurance exercise phase may have had a preconditioning, and even protective effect, thereby influencing the effects of the endurance exercise phase the next day. Preconditioning effects are seen following antioxidant supplementation (Ristow et al. 2009) or with preoperative clamping (Hausenloy and Yellon 2009). The latter activates repair mechanisms in the gut and results in reduced damage from ischemia during the surgery itself. In this study, it was chosen to handle the dual exercise protocol as a single intervention. It would have been also interesting to study the responses evoked by each of the two phases of exercise separately.

### Changes in AA profiles after casein intake

The increase in total plasma AA levels after casein intake was much lower after exercise when compared to the resting condition. As only venous blood was sampled, AA levels may have been influenced by organ uptake and release. Lower levels of total AA levels in plasma could be due to a combination of decreased digestion and absorption in the intestine and more rapid clearance resulting from tissue uptake (e.g., liver and muscle) as source for gluconeogenesis and/or for protein synthesis (Ivy 2004). Casein is able to promote postprandial protein deposition (Boirie et al. 1997), which is beneficial in an exercise condition. Decreased gastric emptying due to the endurance exercise would also lower plasma AA levels. It has, however, been shown that gastric emptying during exercise at the applied intensity occurs at a rate similar (Murray 1987; Carrio et al. 1989) or faster than during rest (Moore et al. 1990), depending on the meal.

The lower plasma total AA levels after exercise were mainly explained by lower levels of nonessential amino acids (nEAA), in particular of alanine. This effect on nEAA levels was not observed in a previous study with resistance‐type exercise, in which mainly the postprandial essential amino acid (EAA) levels were lowered, whereas postprandial nEAA levels were not affected (van Wijck et al. 2013).

A possible explanation for this finding could again lie in the protocol set‐up aimed at glycogen depletion. Gluconeogenesis is an important process during fasting, especially in combination with glycogen depletion, in order to maintain blood glucose levels (Henriksson 1991; Ishikura et al. 2013). The most important AA precursor for gluconeogenesis is alanine, which is also the AA of which systemic levels decrease most rapidly during fasting (Felig et al. 1969). An exception to the decrease in circulating AAs was the higher plasma levels of taurine after exercise compared to those following resting. Taurine can be released upon muscle contraction (Cuisinier et al. 2002; Ishikura et al. 2013), which likely explains this finding.

### Intestinal metabolic capacity

Circulating citrulline is often used as a clinical measure of total intestinal metabolic mass (Crenn et al. 2008). The absence of an increase in circulating citrulline after ingestion of casein following the dual exercise protocol could reflect a reduced intestinal metabolic capacity. Two important AAs in citrulline metabolism are glutamine and arginine, as a precursor and product of citrulline, respectively. Depletion of glutamine, which is the main fuel for enterocytes, results in plasma citrulline depletion (Rougé et al. 2007; Blachier et al. 2009). Findings in mice suggest that during shortage of available glutamine, enterocytes possibly prioritize its utilization for energy over the production of citrulline (Sokolović et al. 2007). Therefore, during periods of energy stress (i.e., fasting and exercise) the glutamine taken up by the intestine may not be converted into citrulline. Our results suggest that after strenuous exercise the intestine's energy stores or metabolic capacity may be insufficient to convert available glutamine from a casein protein bolus into citrulline. This agrees with the parallel decrease in arginine. Metabolic flux studies, with stable isotopes could give more insight in the intestinal uptake and conversion of available glutamine.

In conclusion, we show that the presented dual exercise protocol, combining glycogen depletion and endurance exercise, induces a clear increase in the casein‐derived BCM7 peptide ending up in the urine, independent of paracellular and noncarrier‐mediated intestinal permeability or DPP4 activity. This shows that strenuous exercise could have an effect on the amount of food‐derived bioactive peptides crossing the epithelial barrier. The health consequences of increased passage of these peptides need more in depth studies to determine whether and what effects can occur at the levels found.

## Conflict of Interest

The authors declare that they have no conflict of interest.
